# Toward Sustainable Carbonylation Reactions Enabled by CO Surrogates in Green Solvents

**DOI:** 10.1002/cssc.70797

**Published:** 2026-06-30

**Authors:** Francesco Messa, Serena Perrone, Antonio Salomone

**Affiliations:** ^1^ Dipartimento di Chimica Università degli Studi di Bari “Aldo Moro” Bari Italy; ^2^ Dipartimento di Scienze e Tecnologie Biologiche ed Ambientali Università del Salento Lecce Italy

**Keywords:** carbonyl compounds, carbonylation, CO surrogates, green solvents, metal catalysis

## Abstract

Carbonylation reactions represent a powerful strategy in synthetic chemistry, because they enable the straightforward incorporation of carbonyl functionalities into organic substrates through transition‐metal catalysis in the presence of various nucleophiles. Traditionally, these transformations have been conducted under high pressures of carbon monoxide (CO), a lethal gas, and have relied on volatile organic solvents (VOCs) that are often toxic and derived from petroleum resources, thereby raising significant environmental and safety concerns. In line with the principle of green chemistry, this review summarizes recent advances in carbonylation reactions based on organic and inorganic CO surrogates that safely generate CO on demand within the reaction system, with a specific focus on strategies combining safer CO sources and green solvents classified as more sustainable alternatives to conventional VOCs according to the GSK solvent selection guide.

## Graphical abstract

Carbonylation reactions offer a sustainable route to valuable carbonyl compounds. This review highlights methodologies that exploit carbonylative coupling to drastically enhance process sustainability, avoiding the direct use of CO through CO surrogates and promoting syntheses in green solvents potentially derived from renewable and sustainable sources.

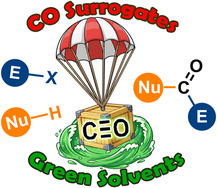

## 1 Introduction

The carbonylation reaction represents an ideal strategy for the development of sustainable syntheses, as it enables the direct incorporation of a carbonyl group into various organic substrates. A distinctive feature of this approach is the ability to minimize the number of steps required for the preparation of highly valuable compounds such as carboxylic acids, esters, amides, and ketones [1, 2, 3, 4].

Following the initial reports by Heck in 1974 [5, 6], this methodology has attracted significant interest, due to its efficiency and versatility, and emerged as a powerful tool for the production of fine chemicals and pharmaceutical ingredients in both academia and industry [7, 8].

Carbonylation reactions are multicomponent processes that are largely aligned with the principles of green chemistry [9, 10, 11], as they enable the one‐pot coupling of at least three components such as a *C*‐electrophile, carbon monoxide, and *N‐*, *O‐*, *C‐*, or *S*‐nucleophile under metal catalysis [12, 13, 14]. This integrated approach affords high atom economy by obviating intermediate isolation and significantly reducing waste generation.

Alongside these undoubtedly advantageous aspects, the originally developed carbonylation methodologies also reveal inherent limitations in terms of sustainability: they rely on the use of carbon monoxide, a toxic and flammable gas that is often employed at high pressures, thereby requiring specialized high‐pressure equipment such as steel autoclaves. Historically, carbonylation reactions have predominantly been performed in toxic, volatile, petroleum‐derived organic solvents, posing serious sustainability challenges from a green chemistry perspective since organic solvents, in fine chemical production, typically account for 50%–80% of the total mass and are responsible for approximately 75% of the cumulative life cycle environmental impact of the entire process [15]. In addition, solvents dominate the toxicity and safety profiles of these processes owing to their volatility, flammability, and potential explosiveness, while also driving the widespread need for personal protective equipment. Furthermore, their nonrenewable origin and limited biodegradability negatively impact resource efficiency and end‐of‐life sustainability, thereby increasing the overall environmental footprint of such processes.

In this review, the choice of synthetic methodologies was guided by the sustainability of the solvents employed, with GSK's solvent selection guide serving as the primary reference [16, 17]. This approach facilitated a systematic assessment of greener solvent options, guided by criteria such as chemical properties, environmental impact, and safety considerations, thereby ensuring that the selected methodologies adhere to the principles of green chemistry and promote sustainable practices.

The development of safe, renewable, and operationally simple carbonylation methodologies has become a central objective for synthetic chemists, especially in the context of large‐scale and industrial applications. Within this framework, the aim of this review is to provide a representative overview of recent advances in carbonylation reactions in which gaseous CO is replaced by organic or inorganic surrogates, and traditional volatile organic solvents are substituted with safer reaction media, such as nonvolatile, environmentally benign solvents potentially obtainable from renewable sources.

Notably, while previous reviews on greener carbonylation reactions have treated CO surrogates [18, 19, 20, 21] and safe solvents [22, 23] as separate strategies, this review intends to highlight methodologies that combine both approaches, producing a synergistic effect in the pursuit of more sustainable carbonylation processes.

The discussion is organized into two main sections according to the CO precursors employed: the first is dedicated to coordination complexes of transition metals (Cr, Fe, Mo, and W) with carbon monoxide, while the second focuses on organic surrogates such as formic acid and its derivatives (formates and formamides), as well as various carboxylic derivatives, including acid chlorides and silacarboxylic acids. Table 1 further provides a concise overview of the carbonylation reactions described in this review, outlining their key experimental parameters (e.g., solvent, catalyst, and CO source) and emphasizing the features that contribute to their high level of sustainability.

**TABLE 1:** Summary of the sustainable carbonylation reactions covered in this review.^a^

Scheme	Products	Solvent	CO source	Catalyst source	Key features
1	EG‐esters	MTPBr/EG	Mo(CO)_6_	Pd(OAc)_2_	Very low Pd loading (0.5 mol%)
3, 4	Amides, esters	ChCl/urea	Mo(CO)_6_	Pd(OAc)_2_	Short reaction time (2 h)
5, 6	Amides, esters	Solvent free	Mo(CO)_6_	Pd(OAc)_2_	Solvent‐free, short reaction time (1.5 h) with ball‐milling
7	Amides, esters	Water or solvent free	Mo(CO)_6_	Fluorinated palladacycle	Very short reaction time (up to 30 min) under microwave irradiation
8	Esters	CPME	Mo(CO)_6_	Pd(OAc)_2_	Sustainable preparation of biologically relevant products
9	*S*‐esters, *Se*‐esters	Anisole	Mo(CO)_6_	Pd(PhCN)_2_Cl_2_	First carbonylative coupling involving selenols
10	Ketones	Anisole	Mo(CO)_6_	Pd(OAc)_2_	Formation of two new C—C bonds
11	Ketones	Anisole	Mo(CO)_6_	Pd‐NHC	Low Pd loading (1.0 mol%)
13	Ketones	Water	Mo(CO)_6_	Fluorinated palladacycle	Short reaction time (up to 30 min) under microwaves
14	Ketones	Anisole	Fe(CO)_5_	Pd—Fe nanocatalyst	Fe modulates the Pd activity (Pd‐loading 1.0 mol%)
15	Ketones	PEG 400	Cr(CO)_6_	PdCl_2_	Carbonylative homocoupling of aryl iodides
16	Acetamides	DMC	W(CO)_6_	Rh_2_(CO)_4_Cl_2_	Aromatic nitro compounds as *N*‐substrates
18	Amides	Water	W(CO)_6_	Pd(OAc)_2_ or palladacycle	Very low Pd loading (0.5—0.8 mol%), micellar medium
19	Ketones	DMSO	HCOOH	Pd(OAc)_2_	Very low energy demand (30°C), synthesis of alkynones
20	Ketones	DMSO	HCOOH	PdCl_2_	Synthesis of α,β‐unsaturated ketones
22	Indolyl methanes	DMSO	TFBen	PdCl_2_(PPh_3_)_2_	In situ formation of aromatic aldehydes
24	1,3‐Thiazolidin‐2‐ones	DMSO	TFBen	*t*‐BuOK	Very low energy demand (35°C), transition metal free
26	1,5‐Dihydro‐2H‐pyrrol‐2‐ones	DMSO	TFBen	Pd(acac)_2_	Double carbonylation with in situ formation of imides
27	Indanones	DMSO	TFBen	Pd(OAc)_2_	Ring expansion of 3‐arylcyclobutanones to 1‐indanones
30	Esters	Propylene carbonate	NFS	Pd/C	Facile catalyst recovery
31	Ketones	Propylene carbonate	NFS	Pd/C	Facile catalyst recovery
32	Ketones	[BMIM][BF_4_] or [BMIM][PF_6_]	NFS	PdCl_2_	Broad applicability to both Suzuki and Hiyama cross‐coupling reactions
34	Ketones	Anisole	COgen^b^	PdCl_2_	Applicable to aryl bromides, preparation of biologically relevant products
35	Amides	α‐Pinene or DMC	COgen^b^	Pd(OAc)_2_	Applicable to aryl bromides, preparation of biologically relevant products
36	Esters	α‐Pinene or 2‐MeTHF or DMC	COgen^b^	Pd(dba)_2_	Applicable to aryl bromides
37	Ketones	Limonene	COgen^b^	Pd(acac)_2_	Applicable to aryl bromides
38	Ketoesters	Anisole	COgen^b^	PdCl_2_	Double carbonylative coupling through a two‐step sequence, applicable to aryl bromides
39	Ketones	Anisole	COgen^b^	PEPPSI‐IPr	Applicable to aryl bromides
40	Thioesters	Anisole or DME	COgen^b^	Pd(OAc)_2_	Preparation of aromatic thioesters
41	Thioesters	Anisole	COgen^b^	PdCl_2_(PhCN)_2_	Thiocarbonylative coupling effective with vinyl, benzyl, and aryl halides
42	Alkynones	Water	SilaCOgen^b^	PdCl_2_(PPh_3_)_2_	Very low energy demand (25°C), water as the solvent
43	Amides	DMC	HCOOH^b^	Pd(PPh_3_)_4_	Effective with aliphatic primary amines
44	Ketones	Anisole	CO_2_	Re(I) bipyridine and PdCl_2_	CO_2_ as source of CO through a photoreduction process
45	Esters	Ethanol	CO_2_	Cathode Cu/Zn and Pd	CO_2_ as source of CO through an electroreduction process

a 2‐MeTHF, 2‐methyl tetrahydrofuran; BMIM, 1‐butyl‐3‐methylimidazolium; ChCl, cholinium chloride; COgen, 9‐methylfluorene‐9‐carbonyl chloride; CPME, cyclopenthyl methyl ether; DMC, dimethylcarbonate; EG, ethylene glycol; MTPBr, methyltriphenylphosphonium bromide; NFS, *N*‐formyl saccharin; PEG, polyethylene glycol; SilaCOgen, 1‐methyl‐1,1‐diphenylsilanecarboxylic acid; TFBen, benzene‐1,3,5‐triyl triformate; PEPPSI‐IPr, [1,3‐Bis(2,6‐Diisopropylphenyl)imidazol‐2‐ylidene](3‐chloropyridyl)palladium(II) dichloride.

b Ex situ generation of CO in a two‐chamber reactor.

## 2 Carbonylations in Sustainable Solvents With Metal Carbonyls as CO Surrogates

To avoid the direct handling of hazardous and toxic carbon monoxide gas and to eliminate the need for specialized equipment, particularly for reactions conducted under pressurized conditions, a wide range of CO surrogates has been developed as alternative carbon monoxide sources. Among these strategies, the in situ generation of CO from transition‐metal carbonyl complexes, Mo(CO)_6_, W(CO)_6_, and Fe(CO)_5_, has emerged as a practical and widely adopted approach [18]. When employed in metal‐catalyzed carbonylation reactions carried out in environmentally benign solvents, these systems markedly improve operational safety while simultaneously enhancing the sustainability and environmental compatibility of carbonylation methodologies.

Among metal carbonyl compounds, molybdenum hexacarbonyl [Mo(CO)_6_] has recently attracted increasing attention to promote carbonylation reactions, due to its ability to provide a safe, practical, and readily handled solid source of carbon monoxide [24]. Carbon monoxide release from Mo(CO)_6_ can be achieved either by thermal activation [25], or through ligand exchange in the presence of Lewis bases, including DBU [26], piperidine [27], and acetonitrile [28].

**SCHEME 1:**
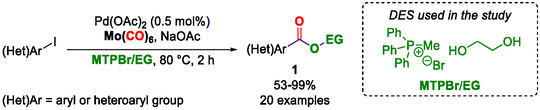
Pd‐catalyzed alkoxycarbonylation of (hetero)aromatic iodides with Mo(CO)_6_ in a phosphonium‐based DES. MTPBr/EG = methyltriphenylphosphonium bromide/ethylene glycol (1/5 mol/mol).

Based on their previous studies on palladium‐catalyzed carbonylative couplings [29, 30, 31], in 2023, Messa et al. reported the first example of a gas‐free Pd‐catalyzed alkoxycarbonylation of (hetero)aryl iodides, by using a nonvolatile and nonflammable phosphonium‐based deep eutectic solvent (DES) [32, 33] as reaction medium and Mo(CO)_6_ as a solid, convenient and easy‐to‐handle source of CO [34]. The combination of a methyltriphenylphosphonium bromide (MTPBr)/ethylene glycol (EG) eutectic mixture as reaction solvent and reagent, Mo(CO)_6_ as CO precursor, NaOAc as green base, mild reaction temperature (80°C), short reaction time (2 h), and a very low catalyst loading [Pd(OAc)_2_, 0.5 mol%], led to a highly efficient synthesis of ethylene glycol esters **1**, obtained in yield up to 99% (Scheme 1).

Moreover, under the optimal reaction conditions and in an open flask, the alkoxycarbonylation of iodobenzene produced the corresponding 2‐hydroxyethyl benzoate in high yield (86%). This result suggests that the CO arising from Mo(CO)_6_ is rapidly transferred from the molybdenum complex to the active palladium species, rather than being released as a gaseous byproduct.

Moreover, using the MTPBr/glycerol (gly) eutectic mixture as the solvent also proved effective for the preparation of glycerol monoesters, although acylation of either the primary or secondary hydroxyl groups of this polyol resulted in the formation of two isomeric products (total yield: 71%–84%).

**SCHEME 2:**
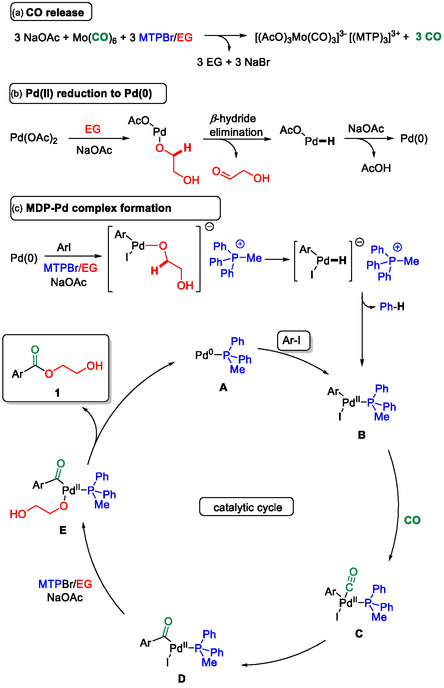
Suggested reaction mechanism for Pd‐catalyzed alkoxycarbonylation of (hetero)aryl iodides with Mo(CO)_6_ in a phosphonium‐based DES. MDP = methyldiphenylphosphine; MTP = methyltriphenylphosphonium; MTPBr/EG = methyltriphenylphosphonium bromide/ethylene glycol.

Regarding the reaction mechanism, the authors proposed that the DES MTPBr/EG not only served as a solvent and source of the nucleophile (EG) for the alkoxycarbonylation coupling but could also play other important roles in the process. In particular, the DES may contribute by enhancing the release of CO from Mo(CO)_6_. Indeed, the eutectic mixture could stabilize, with its organic cation methyltriphenylphosphonium (MTP), the molybdenum tricarbonyl anions formed upon release of three CO equivalents from Mo(CO)_6_ in the presence of the Lewis base NaOAc (Scheme 2a). Moreover, the EG component of the DES could promote the reduction of Pd(II) to Pd(0) (Scheme 2b), and the eutectic mixture could also act as a source of phosphine ligand [methyldiphenylphosphine (MDP)], leading to the formation of MDP–Pd complexes (A–E), thereby modulating the reactivity of palladium (Scheme 2c) [34].

**SCHEME 3:**
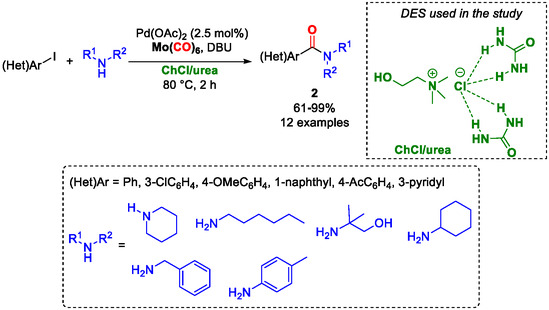
Pd‐catalyzed carbonylation of (hetero)aromatic iodides in the presence of amines as nucleophiles, with Mo(CO)_6_ as safe CO source and in ChCl/urea (1:2 mol/mol) DES as green medium. ChCl = choline chloride; DBU = 1,8‐diazabicycloundec‐7‐ene.

**SCHEME 4:**
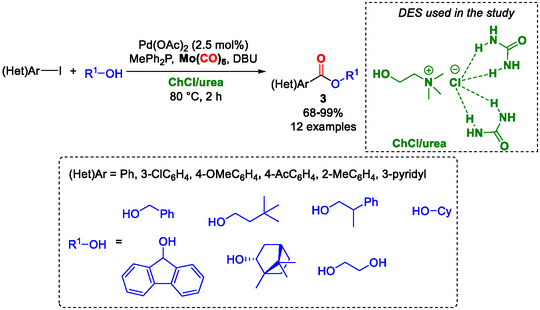
Pd‐catalyzed carbonylation of (hetero)aromatic iodides in the presence of alcohols as nucleophiles, with Mo(CO)_6_ as safe CO source and in ChCl/urea (1:2 mol/mol) DES as green medium. ChCl = choline chloride; DBU = 1,8‐diazabicycloundec‐7‐ene.

To further enhance both the sustainability and applicability of the carbonylative coupling methods in DESs, in 2023, the same authors also reported a Pd‐catalyzed gas‐free synthesis of both aromatic amides and esters, employing choline chloride (ChCl)/urea as a green and bioinspired deep eutectic solvent and Mo(CO)_6_ as a safe source of carbon monoxide [35]. The method afforded a range of carboxylic amides (**2**) in yields of 61%–99% (Scheme 3) and and esters (**3**) in yields of 68%–99% (Scheme 4), respectively. Moreover, the method has been also applied to the gram scale preparation of an active pharmaceutical ingredient (API), the benzyl benzoate, used to treat scabies and lice in humans. The process offers several advantages, including a short reaction time (2 h), a mild reaction temperature (80°C), a low catalyst loading [Pd(OAc)_2_, 2.5 mol%], and the possibility of reusing both the catalyst and the DES for at least four consecutive runs [35].

**SCHEME 5:**
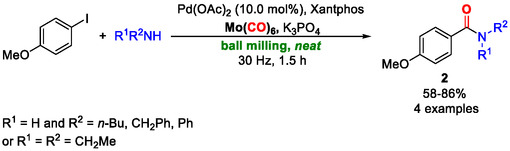
Mechanochemical Pd‐catalyzed carbonylative coupling between 4‐iodoanisole as aromatic iodide and amines as nucleophiles, with Mo(CO)_6_ as safe CO source. XantPhos = 4,5‐bis(diphenylphosphino)‐9,9‐dimethylxanthene.

**SCHEME 6:**
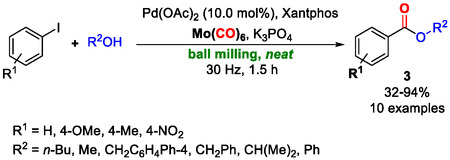
Mechanochemical Pd‐catalyzed carbonylative coupling between aromatic iodides and alcohols as nucleophiles, with Mo(CO)_6_ as safe CO source. XantPhos = 4,5‐bis(diphenylphosphino)‐9,9‐dimethylxanthene.

In 2020, a mechanochemical protocol for Pd‐catalyzed alkoxycarbonylation and aminocarbonylation reactions of aryl iodides, under ball‐milling conditions, was developed [36]. The method employed Mo(CO)_6_ as a solid and convenient carbon monoxide surrogate, thus avoiding the need for direct handling of toxic gaseous CO, as it proved to be the most active of the tested group 6 hexacarbonyl complexes Mo(CO)_6_ (M = Cr, Mo, W). This solvent‐free methodology, using Pd(OAc)_2_ as the catalyst and K_3_PO_4_ as both a Brønsted–Lowry base and a Lewis base to promote CO release from the metal hexacarbonyl via ligand displacement, enabled the synthesis of amides and esters and in yields of 58%–86% and 32%–94%, respectively (Schemes 5 and 6).

Moreover, real‐time monitoring of the mechanochemical model carbonylative coupling between iodobenzene and *n*‐butanol as nucleophile, performed via in situ pressure sensing, suggested that CO insertion into the catalytically active palladium species occurs rapidly and directly from Mo(CO)_6_, with no appreciable release of molecular carbon monoxide [36].

**SCHEME 7:**
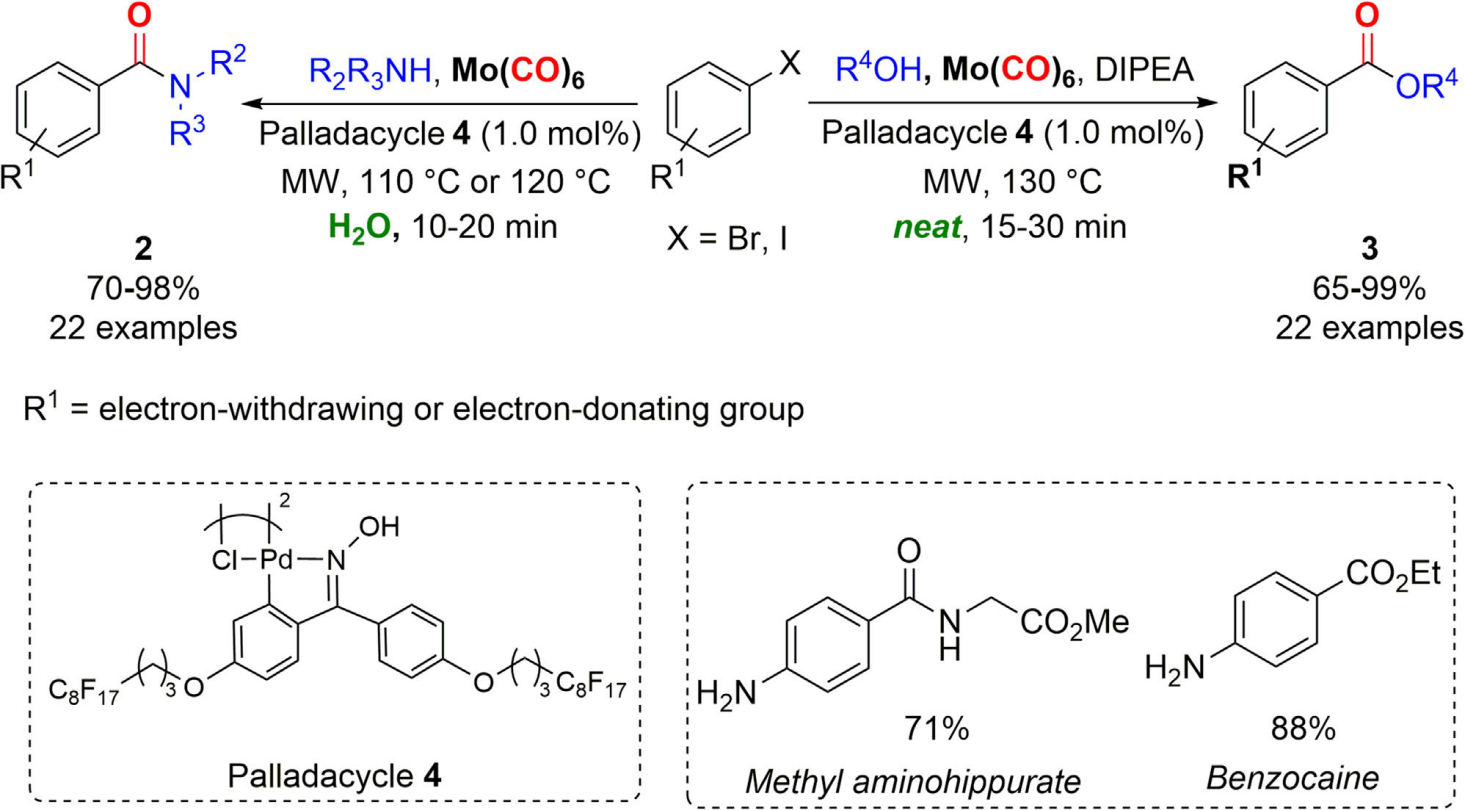
Microwave‐assisted amino and alkoxycarbonylations of aryl halides with amines and alcohols in aqueous medium or under neat conditions, using Mo(CO)_6_ as solid CO source. DIPEA = *N*,*N*‐diisopropylethylamine.

Among the most notable and sustainable carbonylation methodologies, Lam and Lo reported a versatile microwave‐assisted carbonylation of aryl halides using alcohols or amines as nucleophiles, performed in aqueous medium or under neat conditions [37]. Thanks to the synergy between Mo(CO)_6_ as a CO solid source, a fluorinated oxime‐based palladacycle **4** as the catalyst, microwave irradiation, moderate temperatures (110°C for aryl iodides and 120°C for aryl bromides), and aqueous solvent, the aminocarbonylation of a broad range of aryl iodides with aromatic and aliphatic amines afforded amides **2** in yields of up to 98% (Scheme 7). Likewise, the alkoxycarbonylation of diverse aryl and heteroaryl iodides and bromides with alkyl or aryl alcohols proceeded efficiently under solvent‐free conditions, delivering esters **3** in yields of up to 99% (Scheme 7) [37].

Furthermore, the catalytic system **4** exhibited an excellent recyclability over five consecutive cycles, showing a little loss of catalytic activity and a low level of Pd leaching. In addition, both the alkoxy‐ and aminocarbonylation protocols were successfully applied to the synthesis of pharmaceutically relevant molecules, including the diagnostic agent methyl aminohippurate and the local anesthetic benzocaine (Scheme 7) [37].

**SCHEME 8:**
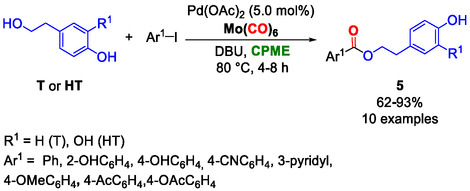
Pd‐catalyzed carbonylation of (hetero)aromatic iodides in the presence of the alcohols **T** or **HT** as nucleophiles, with Mo(CO)_6_ as safe CO source and in CPME as eco‐friendly medium. CPME = cyclopentyl methyl ether; DBU = 1,8‐diazabicycloundec‐7‐ene; **HT** = hydroxytyrosol; **T** = tyrosol.

In 2025, the metal carbonyl Mo(CO)_6_ was also employed as a solid surrogate, replacing hazardous gaseous carbon monoxide, in an eco‐friendly Pd‐catalyzed alkoxycarbonylation process for the regioselective synthesis of tyrosol (**T**) and hydroxytyrosol (**HT**) esters [38]. The carbonylative couplings between aromatic iodides and the naturally occurring alcohols T or HT, were carried out under mild conditions (80°C), in short reaction times (4–8 h), using the sustainable and biomass‐derived solvent cyclopentyl methyl ether (CPME) [39]. These reactions furnished T and HT ester derivatives 5 in 62%–93% yield (Scheme 8). Additionally, all synthesized compounds exhibited notable antioxidant properties and some benzoic ester derivatives were able to inhibit the enzyme α‐glucosidase in the micromolar range, indicating potential therapeutic relevance for the management of hyperglycemia [38].

**SCHEME 9:**
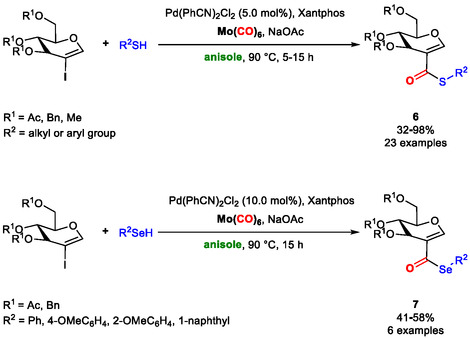
Thio‐ and seleno‐carbonylation of 2‐iodoglycals in the presence of Mo(CO)_6_ as convenient CO source and in anisole as green medium.

In 2020, Stefani and coworkers developed a sustainable Pd‐catalyzed approach for the synthesis of thioesters and selenol esters bearing glycals, via a thio‐ and seleno‐carbonylation of 2‐haloglycals. Specifically, various *O*‐protected‐2‐iodoglycals were reacted with thiols and selenols, in the presence of Mo(CO)_6_ as a carbon monoxide source and anisole [16] as an environmentally benign solvent (Scheme 9) [40]. Under heating at 90°C for 5–15 h, the procedure allowed the preparation of C2‐glycosides **6**, bearing a thioester functional group, in yields up to 98%, and with high functional‐group tolerance. The lowest yield of 32% was achieved with the use of 2‐aminobenzenethiol as nucleophile, probably because of the chelation of this substrate to palladium catalyst. The scope of the protocol was further extended to selenols, providing access to six glycal‐derived selenol esters **7**. Although this work represents a milestone as the first example of a carbonylative cross‐coupling reaction involving selenols, the desired products **7** were obtained in only moderate yields (41%–58%, Scheme 9) indicating that further optimization is still required [40].

Given the increasing interest in metal‐catalyzed C—C bond‐forming reactions, three‐component carbonylative cross‐coupling reactions of aryl or heteroaryl halides with a metal carbonyl as a convenient CO source and arylboronic acids has emerged also as an effective strategy for constructing diaryl ketone scaffold. Diaryl ketones are important structural motifs across various fields and represent key intermediates in numerous organic transformations [41].

**SCHEME 10:**
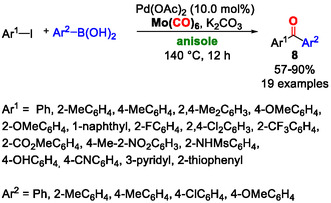
Carbonylative arylation of aryl and heteroaryl iodides with Mo(CO)_6_ as safe CO source and in anisole as green medium. Ms = methyl sulfonyl group.

For example, a broad range of iodoarenes bearing electron‐withdrawing or electron‐donating substituents, together with various arylboronic acids, were used as substrates in a versatile Pd‐catalyzed carbonylative arylation to access unsymmetrical diaryl ketones (**8**, Scheme 10) [42]. This Suzuki–Miyaura carbonylative cross‐coupling employed Mo(CO)_6_ as a convenient, thermally activated CO source, releasing carbon monoxide in situ at 140°C, K_2_CO_3_ as a base, and anisole as a greener reaction medium. Under these conditions, the reaction furnished the corresponding diaryl ketones in moderate to excellent yields (57%–90%, Scheme 10) [42].

**SCHEME 11:**
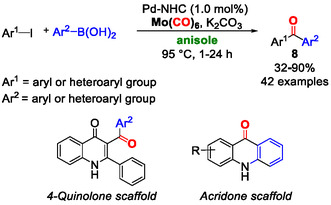
Carbonylative Suzuki reaction between arylboronic acids and aryl iodides with Mo(CO)_6_ as safe CO source and in anisole as green medium. Pd–NHC = palladium–*N*‐heterocyclic carbene complexes.

The same combination of K_2_CO_3_ as the base, anisole as an eco‐friendly solvent and Mo(CO)_6_ as a safe CO surrogate, revealed to be the best choice also for the carbonylative Suzuki reaction between arylboronic acids and aryl iodides, as described by Das and coworkers in 2017 (Scheme 11) [43]. Compared to the protocol described in Scheme 10, this methodology enabled C—C bond formation in the presence of a lower loading of Pd catalyst (Pd–NHC, 1.0 mol%) and a milder reaction temperature (95°C). The procedure exhibited a broad substrate scope and tolerated a range of functional groups (including –COMe, –COOMe, –NH_2_, –CN, –F, –Cl, and –Br), delivering unsymmetrical diaryl ketones in 32%–90% yield. Furthermore, the carbonylative coupling was successfully applied to the synthesis of biologically relevant 4‐quinolone and acridone scaffolds (Scheme 11) [43].

**SCHEME 12:**
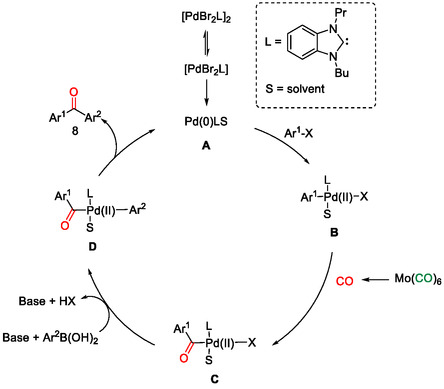
Suggested reaction mechanism for carbonylative Suzuki reaction between arylboronic acids and aryl iodides.

About the reaction mechanism, it has been suggested that the active Pd(0) species **A**, in the presence of the aryl iodide, could undergo oxidative addition to produce the aryl–palladium intermediate (**B**). Subsequent CO insertion into the Pd—C(aryl) bond could yield the acylpalladium complex **C**. A following transmetallation reaction with the boronic acid could form the intermediate **D**, which then could undergo reductive elimination to furnish the desired product **8**, simultaneously regenerating the Pd(0) catalyst (Scheme 12) [43].

**SCHEME 13:**
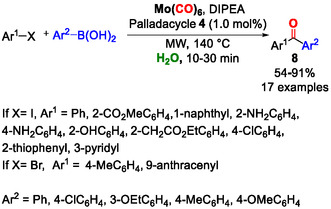
Microwave‐assisted carbonylative Suzuki–Miyaura cross‐coupling using water as solvent and Mo(CO)_6_ as safe CO source. DIPEA = *N*,*N*‐diisopropylethylamine.

To ensure the environmental sustainability of the process, Lam and Lo's group developed a microwave‐assisted carbonylative Suzuki–Miyaura cross‐coupling performed in aqueous medium and catalyzed by a fluorous oxime‐based palladacycle **4** (see Scheme 7) [37]. At 140°C, Mo(CO)_6_ undergoes thermal decomposition to generate carbon monoxide in situ, which then participates in the carbonylative coupling of aryl (or heteroaryl) halides with arylboronic acids, bearing electron‐donating or electron‐withdrawing substituents. The corresponding diaryl ketones were obtained in moderate to good yields (54%–91%, Scheme 13) [37].

**SCHEME 14:**
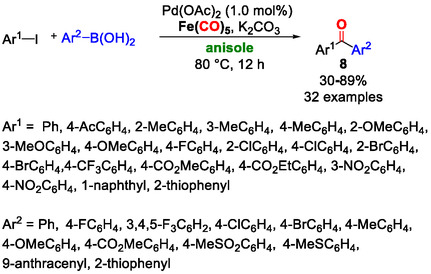
Carbonylative Suzuki reaction between arylboronic acids and aryl iodides, using Fe(CO)_5_ as safe CO source and anisole as sustainable solvent.

Although Mo(CO)_6_ is the most commonly used metal carbonyl for in situ CO generation in sustainable carbonylative cross‐coupling reactions, only a few studies have reported the use of alternative metal carbonyls. For example, diaryl ketones can also be prepared via Pd‐catalyzed carbonylative Suzuki–Miyaura cross‐couplings, employing iron(0) pentacarbonyl Fe(CO)_5_ as a safer CO releasing surrogate and anisole as a sustainable solvent, under mild conditions (Scheme 14) [44]. In the protocol, developed by Gao's group in 2021, the transition metal carbonyl Fe(CO)_5_ plays a dual role: it provides CO in situ for the carbonylation, and the resulting iron residues modulate the catalytic behavior of palladium through the in situ formation of a more active Pd–Fe nanocatalyst. The scope and limitations of the methodology were assessed using a variety of iodoarenes and boronic acids, bearing both electron‐withdrawing and electron‐donating groups on the aryl substituent. The synthetic procedure showed a wide functional‐group tolerance, affording diaryl ketone derivatives **8** in yields up to 89% (Scheme 14) [44].

Moreover, the bimetallic Pd–Fe nanoparticles, whose structural features were characterized by TEM, SEM, EDX, XPS, and FT‐IR analyze, can be recycled in multiple reaction cycles. In particular, for the carbonylation of 4‐iodoacetophenone with phenylboronic acid to afford the corresponding benzophenone, the isolated catalyst, when supplemented with fresh Fe(CO)_5_, could be reused for up to four consecutive runs, with product yields decreasing from 90% to 50% [44].

**SCHEME 15:**
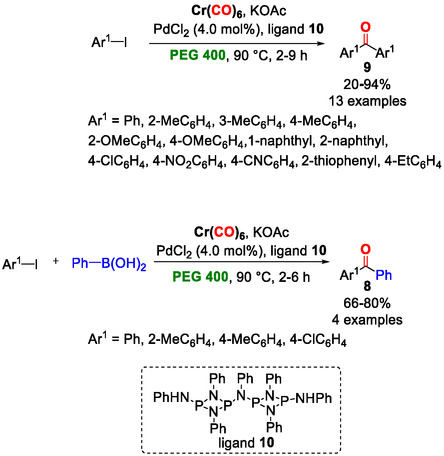
Synthesis of symmetrical and asymmetrical diaryl ketones via carbonylative couplings of aryl iodides with Cr(CO)_6_ as practical CO source and PEG 400 as eco‐friendly solvent.

In 2016, Etemadi–Davan and Iranpoor developed an efficient Cr(CO)_6_‐mediated carbonylative homocoupling of aryl iodides to deliver symmetrical diaryl ketones **9** in yields ranging from 20% to 94% (Scheme 15) [45]. The procedure employed PdCl_2_ as the catalyst, a dinuclear 1,3,2,4‐diazadiphosphetidine oligomer ([(PhNH)P_2_(NPh)_2_]_2_NPh, **10**) as a cheap and heterogeneous palladium bidentate ligand, Cr(CO)_6_ as a practical CO source, and PEG 400 as a green solvent [46] (Scheme 15). The methodology showed broad functional‐group tolerance, efficiently converting substrates bearing both electron‐donating and electron‐withdrawing substituents into the corresponding symmetrical diaryl ketones in high yields. A significant decrease in yield was observed only for substrates containing –NO_2_ and –CN groups (25% and 20%, respectively), likely due to the reducibility of the nitro group and the potential coordination of the cyano group to chromium carbonyl species.

Moreover, the applicability of this transformation was further extended for unsymmetrical diaryl ketones preparation. Indeed, similar experimental conditions were also applied to perform Suzuki–Miyaura couplings between iodoarenes and phenyl boronic acid, obtaining the corresponding ketones **8** in yield up to 80% (Scheme 15) [45].

**SCHEME 16:**
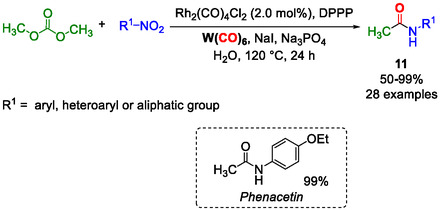
Rhodium‐catalyzed aminocarbonylation reaction for the synthesis of acetamides, with dimethyl carbonate (DMC) both as a C1 building block and as a green reaction medium and W(CO)_6_ as CO source.

Tungsten hexacarbonyl, W(CO)_6_, represents another metal carbonyl employed as a carbon monoxide surrogate in carbonylative cross‐coupling reactions. In 2021, Wu and coworkers showed a novel and sustainable rhodium‐catalyzed aminocarbonylation protocol for the synthesis of acetamides (**11**, Scheme 16) [47]. This carbonylative transformation exploits dimethyl carbonate (DMC) both as a C1 building block and as a green reaction medium [48], in combination with aliphatic and aromatic nitro compounds. Notably, W(CO)_6_ plays a dual role in the process, functioning both as a carbon monoxide source and as a reductant for the in situ formation of amines from the corresponding nitro compounds.

Under the optimized reaction conditions, employing Rh_2_(CO)_4_Cl_2_ as the catalyst, 1,3‐bis(diphenylphosphino)propane (DPPP) as ligand, NaI as a cocatalyst, Na_3_PO_4_ as base, and W(CO)_6_ in the presence of water at 120°C for 24 h, the aminocarbonylation proceeded efficiently, affording the corresponding acetamides **11** in moderate to excellent yields (50%–99%, Scheme 16). Moreover, to further improve the potential utility of this carbonylative cross‐coupling, it was applied to the preparation of the nonsteroidal anti‐inflammatory drug phenacetin, obtained in almost quantitative yield starting from DMC and the 1‐ethoxy‐4‐nitrobenzene (Scheme 16) [47].

**SCHEME 17:**
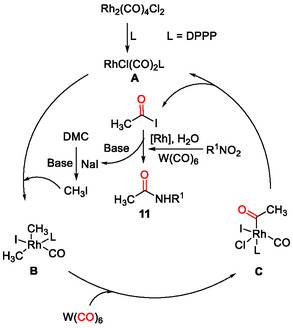
Proposed mechanism for the synthesis of acetamides via rhodium‐catalyzed aminocarbonylation reaction of DMC. DPPP = 1,3‐bis(diphenylphosphino)propane.

Based on the proposed reaction mechanism, NaI plays a key role in the overall transformation. It was suggested to promote the conversion of DMC into methyl iodide, which could undergo oxidative addition to the catalytically active rhodium species **A**, generating intermediate **B**. Subsequently, the amine formed in situ from the reduction of the nitro compound could act as a nucleophile toward the acetyl iodide intermediate, leading to the formation of acetamide derivative **11** (Scheme 17) [47].

**SCHEME 18:**
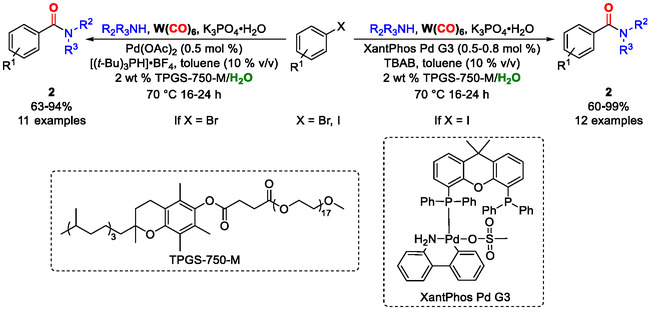
Pd‐catalyzed aminocarbonylation reaction of aryl/heteroaryl halides in aqueous micellar solvent using W(CO)_6_ as practical alternative CO source.

The metal carbonyl complex W(CO)_6,_ used as a practical alternative CO source, obviated the need for specialized equipment and direct handling of gaseous CO even in an efficient Pd‐catalyzed aminocarbonylation of halides carried out in aqueous micellar medium, as described by Lipshutz et al. in 2023 [49]. This versatile and green approach involved a wide range of both aryl/heteroaryl iodides and bromides, as well as both primary or secondary amines as nucleophiles, leading to the corresponding amides with high functional‐group tolerance. Under the optimized conditions, the use of the surfactant TPGS‐750‐M in combination with toluene as a cosolvent proved essential, delivering the target amide products (**2**) in yields ranging from 60% to 99% (Scheme 18).

The environmental impact of the process was assessed through calculation of the E‐factor, which was determined to be 8.7 when including the extraction solvent and 3.9 when excluding it, underscoring the low waste generation associated with this protocol. Notably, the versatility of the methodology was further demonstrated in the synthesis of relevant amide‐containing APIs. The anticancer agent sonidegib was obtained in 62% overall yield over two steps, while a bitopertin analog was prepared through a three‐step, one‐pot sequence in 51% yield [49].

## 3 Carbonylations in Sustainable Solvents With Organic CO Surrogates

### 3.1 Formic Acid and Its Derivatives as In Situ CO Sources

To avoid the hazards associated with handling toxic gaseous carbon monoxide, along with a number of metal carbonyls, organic CO‐releasing precursors have also been developed and applied to carbonylation chemistry. Representative examples include formic acid and its derivatives, acid chlorides, and aldehydes, among others [18, 21].

In carbonylation reactions, formic acid can serve as an in situ CO precursor. Under acidic and high‐temperature conditions, formic acid undergoes thermal decomposition to generate carbon monoxide and water. Alternatively, CO can be formed at lower temperatures through the reaction of formic acid with a suitable activator such as acetic anhydride (Ac_2_O) or dicyclohexylcarbodiimide (DCC) [18]. In the first case, the HCOOH/Ac_2_O system produces acetic acid as a byproduct, which can partially neutralize the base required for the carbonylative process. By contrast, DCC is good dehydrating that efficiently extracts water from formic acid and promotes the release of CO, thereby enabling carbonylation under milder conditions [18].

**SCHEME 19:**
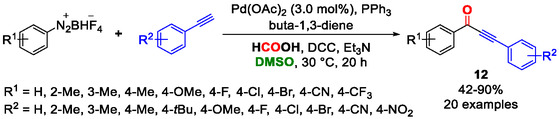
Pd‐catalyzed carbonylative Sonogashira with formic acid as the CO precursor and DMSO as solvent. DCC = dicyclohexylcarbodiimide.

In 2017, Wu and Peng [50] developed an effective Pd‐catalyzed carbonylative Sonogashira employing aryl diazonium salts and terminal alkynes as coupling partners, formic acid as the CO surrogate, DCC as its activator, and DMSO as an eco‐friendly solvent [16]. The methodology exhibited broad functional‐group tolerance on both coupling components and proceeded under mild conditions in the presence of 1,3‐butadiene, to afford a range of alkynone derivatives **12** in moderate to good yields (42%–90%, Scheme 19). Alkynones constitute valuable synthetic intermediates [51] and structural motifs present in numerous bioactive molecules [52]. Notably, the presence of the low‐hindered 1,3‐diene (1,3‐butadiene) proved crucial for the efficiency of the transformation, presumably due to its insertion into the palladium coordination sphere, thereby promoting the carbonylation process. In its absence, only low yields of the desired carbonylated products were obtained [50].

**SCHEME 20:**
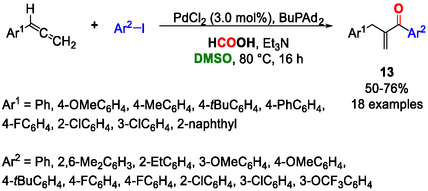
Palladium‐catalyzed carbonylative coupling of aryl iodides and arylallenes, with formic acid as the CO source, and DMSO as the optimal solvent. BuPAd_2_ = di(1‐adamantyl)*‐N*‐butylphosphine.

In 2020, Wu and coworkers described the synthesis of α‐branched enones, a valuable class of *α*,β‐unsaturated ketones [53]. Specifically, enones **13** were prepared via a palladium‐catalyzed carbonylative coupling reactions of aryl iodides and arylallenes (Scheme 20). In this protocol, DMSO proved to be the optimal solvent and formic acid was employed as a convenient CO precursor and organic reductant. The desidered products **13** were obtained in moderate to good yields after 16 h at 80°C with PdCl_2_/BuPAd_2_ as the catalyst system. Several aryl iodides and allenes were tested showing that the protocol was compatible with different mono and disubstituted iodobenzenes, as well as with phenylallenes having electron‐withdrawing or electron‐donating substituents on the aromatic ring, thus demonstrating good functional‐group tolerance (Scheme 20) [53].

Among the derivatives of formic acid, in 2016, Wu's group reported for the first time benzene‐1,3,5‐triyl triformate (TFBen) as a solid, convenient, and efficient CO surrogate capable of promoting numerous carbonylation reactions [54].

**SCHEME 21:**
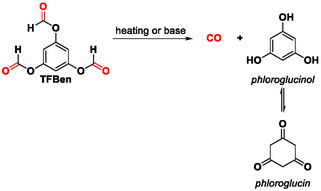
TFBen (benzene‐1,3,5‐triyl triformate) as a CO surrogate.

Notably, TFBen readily releases carbon monoxide upon heating or under basic conditions (Scheme 21), while generating phloroglucinol (1,3,5‐trihydroxybenzene) as a noninterfering byproduct. Indeed, due to its symmetric arene substitution pattern, phloroglucinol exists in solution in equilibrium with its keto tautomer 1,3,5‐cyclohexanetrione (phloroglucin) that makes it less or unreactive toward metal catalyst intermediates. For these reasons, TFBen is considered a potent and nonreacting solid CO source [54].

**SCHEME 22:**
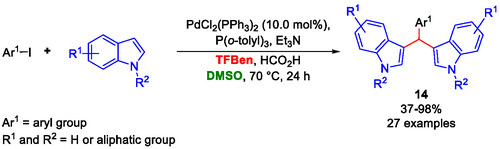
Synthesis of bis(indolyl)methanes in DMSO using TFBen as a convenient CO source.

Bis(indolyl)methanes represent important structural motifs found in natural products, pharmaceuticals, and other biologically active compounds [55, 56]. In 2018, Wu and coworkers described an efficient palladium‐catalyzed carbonylative synthesis of bis(indolyl)methanes **14** starting from aryl iodides and indoles (Scheme 22) [57]. Using PdCl_2_(PPh_3_)_2_ as the optimal catalyst in combination with a phosphine ligand, Et_3_N as the base, formic acid, DMSO as an eco‐friendly solvent [16], and TFBen as a solid CO surrogate, a broad range of bis(indolyl)methane derivatives **14** were obtained in moderate to excellent yields (37%–98%, Scheme 22). Both electron‐rich and electron‐deficient aryl iodides were compatible with the protocol, although electron‐poor substrates generally provided lower yields. In addition, the reaction tolerated both *N*H‐free and *N*‐substituted indoles, delivering the target products in good yields.

**SCHEME 23:**
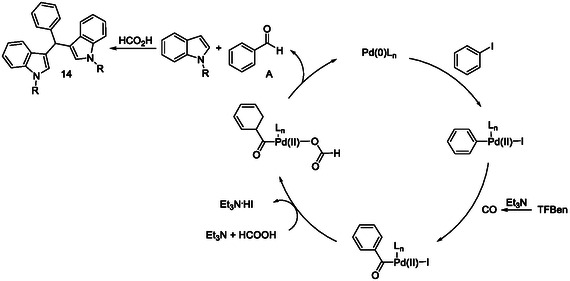
Suggested mechanism for the synthesis of bis(indolyl)methanes **14**.

On the basis of their experimental findings, the authors proposed a mechanism in which the aryl iodide undergoes reductive carbonylation to generate the corresponding aryl aldehyde **A** in situ (Scheme 23). This intermediate then undergoes formic acid‐promoted nucleophilic addition by the indole to furnish the bis(indolyl)methane product **14**. Notably, no product formation was observed in the absence of formic acid. The presence of formic acid thus appears to play multiple crucial roles in the catalytic process: (1) serving as the proton source required for the reductive carbonylation step leading to the benzaldehyde intermediate **A**, (2) promoting the subsequent reaction between the benzaldehyde and indole to give the final product, and (3) shifting equilibria to avoid undesired nucleophilic attack of *N*H‐free indoles on the acyl–Pd(II) intermediate [57].

**SCHEME 24:**
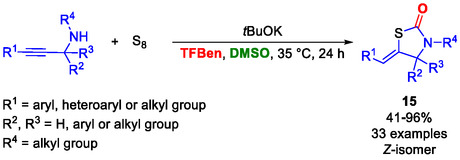
Synthesis of 1,3‐thiazolidin‐2‐ones in DMSO using TFBen as a solid CO precursor.

The combination of DMSO as solvent and TFBen as a benign, solid CO source was also exploited by Wu and coworkers for the synthesis of 1,3‐thiazolidin‐2‐ones [58], a family of sulfur‐containing heterocycles present in numerous bioactive compounds [59]. In particular, the authors developed a *t*BuOK‐mediated carbonylative protocol involving the cyclization of propargylic amines with elemental sulfur, which furnished a series of substituted 4‐alkylidene‐thiazolidin‐2‐ones **15** in 41%–96% yields. Notably, the process proceeds under mild and metal‐free conditions and exhibits broad functional‐group tolerance (Scheme 24) [58].

**SCHEME 25:**
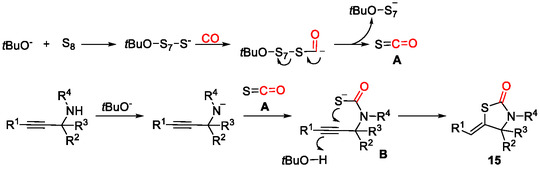
Proposed reaction mechanism for the synthesis of 1,3‐thiazolidin‐2‐ones **15**.

A plausible reaction mechanism was proposed in which carbonyl sulfide (S=C=O) (**A**, Scheme 25), generated in situ upon reaction of *t*BuO^−^ with elemental sulfur (S_8_), could act as a key intermediate. Nucleophilic attack by the deprotonated propargylic amine on this species could form the corresponding thiocarbamate intermediate **B**, which then could undergo intramolecular cyclization to deliver the desired sulfur‐containing heterocyclic product **15** (Scheme 25) [58].

Building on the promising results obtained for the carbonylative cyclization of propargylic amines with elemental sulfur in DMSO using TFBen as a solid CO surrogate, in 2020, Wu and coworkers further explored the catalytic carbonylation of propargylic amines with aryl and vinyl halides [60].

**SCHEME 26:**
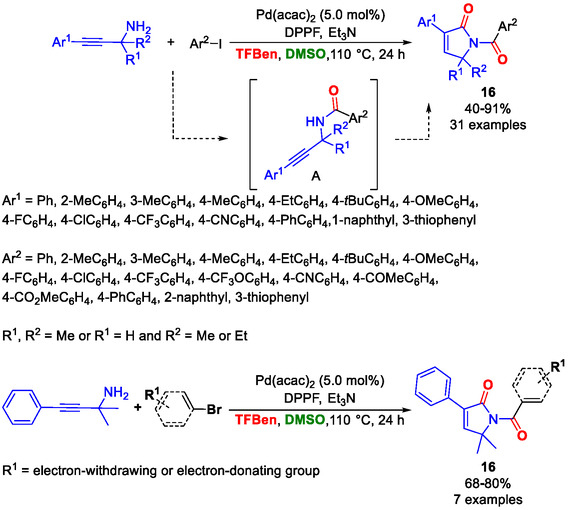
Pd‐catalyzed synthesis of 1‐aroyl‐3‐aryl‐1,5‐dihydro‐2H‐pyrrol‐2‐ones from propargyl amines and aryl or vinyl halide, in DMSO using TFBen as a solid CO source. DPPF = 1,1′‐bis(diphenylphosphino)ferrocene; Pd(acac)_2_ = palladium(II)acetylacetonate.

The reaction, performed under the optimal reaction conditions shown in Scheme 26, enabled a Pd‐catalyzed double carbonylation to a series of 1‐aroyl‐3‐aryl‐1,5‐dihydro*‐2H*‐pyrrol‐2‐ones **16** in 40%–91% yield. Notably, the 2‐oxo‐2,5‐dihydropyrrole scaffold constitutes a valuable structural motif encountered in numerous biologically and pharmacologically active molecules [61, 62]. Both aryl iodides with electron‐withdrawing groups and with electron‐donating substituents were successfully converted to the expected products **16**, as well as the reaction tolerated a broad range of propargylic amines. A linear monocarbonylated amide intermediate **A** was proposed as the key species in this double carbonylation process (Scheme 26).

Moreover, the protocol could be extended to aryl and vinyl bromides, which furnished the corresponding products **16** in moderate to good yields (68%–80%, Scheme 26) [60].

**SCHEME 27:**
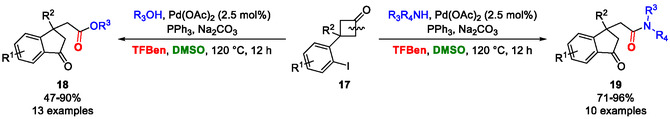
Pd‐catalyzed carbonylative synthesis of indanones, in DMSO using TFBen as a convenient CO surrogate.

Recently, the combination of DMSO as solvent and TFBen as a benign solid CO surrogate was further exploited by Cao and Xu to access a broad range of indanone derivatives bearing ester or amide functionalities [63], valuable structural units commonly found in natural products and pharmaceutical molecules [64, 65]. In particular, indanone esters **18** and indanone amides **19** were synthesized via a Pd‐catalyzed carbonylative C—C bond activation of cyclobutanones **17**, involving C—C bond cleavage, ring opening, and subsequent amino‐ or alkoxycarbonylation (Scheme 27).

Under the optimized conditions, TFBen as the CO source, Pd(OAc)_2_ (2.5 mol%) as the catalyst, PPh_3_ as the ligand, and Na_2_CO_3_ as the base in DMSO at 120°C, the desired products **18** and **19** were obtained in 47%–90% and 71%–96% yield, respectively (Scheme 27). The protocol showed good compatibility with a variety of substituted cyclobutanones, as well as with primary aliphatic alcohols and with primary and secondary alkyl amines. In contrast, secondary/tertiary alcohols and aryl amines delivered significantly reduced yields or remained unreactive [63].

Aryl formates were also competent reagents in this transformation, to furnish the corresponding phenyl esters. In this context, aryl formates played a dual role: in the presence of a base, they acted as CO surrogates and simultaneously generated phenols *in* situ, which then served as nucleophiles. In contrast, alkyl formates failed to deliver the desired products. Furthermore, when aliphatic alcohols were used as external nucleophiles in the presence of phenyl formate as the CO source, only the phenyl ester was obtained, likely due to the higher nucleophilicity of the in situ formed phenol with respect to aliphatic alcohols. Indeed, TFBen was identified as the CO surrogate of choice for this carbonylative protocol [63].

**SCHEME 28:**
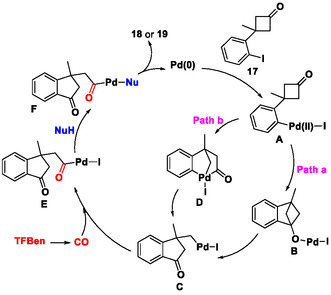
Proposed mechanism for the Pd‐catalyzed carbonylative synthesis of indanones.

A plausible reaction mechanism involves initial oxidative addition of Pd(0) to the C—I bond of cyclobutanone **17** to afford aryl–Pd species **A**. Intermediate **A** may then undergo intramolecular nucleophilic addition to the carbonyl group followed by β‐carbon elimination to generate σ‐alkylpalladium intermediate **C** (Path a, Scheme 28). Alternatively, a second oxidative addition of species **A** could furnish a Pd(IV) intermediate **D**, with subsequent reductive elimination leading to species **C** (Path b, Scheme 28). Insertion of CO derived from TFBen into intermediate **C** would then afford acylpalladium species **E**, which subsequently reacts with the nucleophile (alcohol or amine) to deliver the ester **18** or amide **19** products, while regenerating the Pd(0) catalyst (Scheme 28) [63].

Given the safety concerns associated with handling gaseous carbon monoxide, the development of CO releasing molecules has attracted considerable attention over the past decades, and numerous CO surrogates have been explored for use in carbonylative transformations.

**SCHEME 29:**
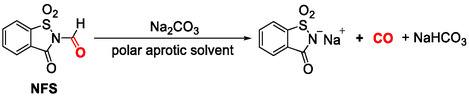
Decarbonylation of *N*‐formylsaccharin (NFS).

Among formic acid‐derived reagents, *N*‐formylsaccharin (NFS), an easily accessible crystalline compound commonly employed as a formylating agent [66], was introduced as a CO surrogate by Manabe's research group in 2013, in the context of a palladium‐catalyzed reductive carbonylation of aryl bromides [67]. In polar aprotic solvents such as *N*,*N*‐dimethylformamide [67] or dichloromethane [68] and in the presence of mild bases (such as NEt_3_ or Na_2_CO_3_), NFS undergoes rapid decarbonylation at room temperature, releasing CO and generating saccharinate as a benign crystalline byproduct within 30 min (Scheme 29) [67]. Notably, the saccharinate generated as a side product can be readily separated from the reaction mixture and recycled for the synthesis of *N*‐formylsaccharin [66]. Despite the efficiency of this process, the use of the aforementioned amide and chlorinated solvents poses sustainability and safety concerns. In contrast, organic cyclic carbonates, such as ethylene carbonate and propylene carbonate, have emerged as environmentally benign polar aprotic solvents, owing to their low toxicity, biodegradability, low vapor pressure, and derivation from renewable feedstocks [69].

**SCHEME 30:**
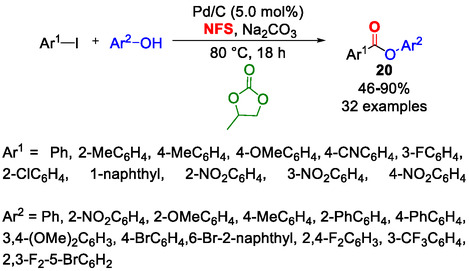
Pd‐catalyzed phenoxycarbonylation of aryl iodides using NFS as CO surrogate and propylene carbonate as sustainable solvent. NFS = *N*‐formylsaccharin.

In this contest, and based on the relatively low nucleophilicity of saccharin byproduct compared to phenols, Bhanage and coworkers in 2017 exploited the first phenoxycarbonylation reaction employing NFS as a readily accessible and highly reactive CO surrogate and propylene carbonate as an environmentally benign polar aprotic solvent [70]. Particularly, a Pd‐catalyzed carbonylative cross‐coupling between aryl iodides and phenols was developed, affording a structurally diverse set of phenyl esters **20** in moderate to excellent yields (46%–90%, Scheme 30).

In this protocol, NFS served as a convenient crystalline CO precursor, while Na_2_CO_3_ proved optimal both for promoting NFS decarbonylation and for driving the carbonylative coupling in propylene carbonate. The use of heterogeneous Pd/C further enhanced the environmental profile of the method, enabling facile catalyst recovery and reuse over four consecutive runs without significant loss of activity. Taken together, these features render this NFS‐based phenoxycarbonylation an operationally simple, efficient, and environmentally benign approach for the synthesis of aromatic esters [70].

**SCHEME 31:**
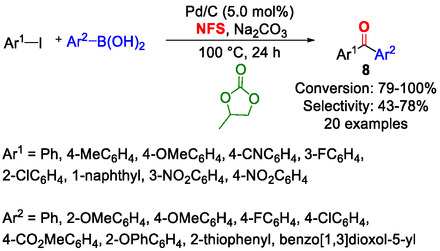
Pd/C‐catalyzed carbonylative Suzuki–Miyaura cross‐couplings using NFS as CO surrogate and propylene carbonate as sustainable solvent. NFS = *N*‐formylsaccharin.

Moreover, *N*‐formylsaccharin/propylene carbonate combination was further extended to Pd/C‐catalyzed carbonylative Suzuki–Miyaura cross‐couplings of aryl iodides with aryl boronic acids to provide a variety of biaryl ketones (Scheme 31) [71]. The protocol displayed a broad substrate scope, tolerating aryl iodides bearing both electron‐donating and strongly electron‐withdrawing substituents (e.g., *meta*‐fluoro, *para*‐cyano, *meta*‐nitro, and *para*‐nitro groups), as well as a range of differently substituted aryl boronic acids, affording the corresponding ketones **8** in moderate to good conversions and selectivities after 24 h at 100°C (Scheme 31). Consistent with the phenoxycarbonylation protocol, the use of heterogeneous Pd/C enabled efficient catalyst recovery and reuse, with the catalyst remaining active over up to five consecutive cycles with only marginal loss of performance [71].

**SCHEME 32:**
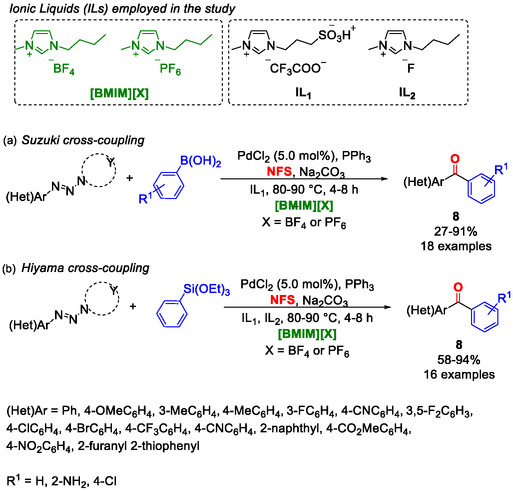
Carbonylative Suzuki and Hiyama cross‐couplings using NFS as CO source and ionic liquids (ILs) as sustainable solvents. NFS = *N*‐formylsaccharin.

More recently, *N*‐formylsaccharin (NFS) has been demonstrated to be a robust and versatile CO surrogate in the synthesis of diaryl ketones via carbonylative Suzuki–Miyaura and carbonylative Hiyama cross‐coupling reactions. In this protocol, aryl triazenes were employed as alternative aryl electrophiles in place of conventional aryl halides, reacting with phenylboronic acids or triethoxy(phenyl)silane, respectively (Scheme 32) [72]. These palladium‐catalyzed transformations were performed in imidazolium‐based ionic liquids (ILs), which were selected as attractive media for the development of more sustainable carbonylative processes [73]. In particular, [BMIM][BF_4_] or [BMIM][PF_6_] served as green solvents, in combination with IL_1_ or IL_2_ as reaction promoters (Scheme 32). Under the optimized conditions, employing PdCl_2_/PPh_3_ as the catalytic system, the desired diaryl ketones **8** were obtained in moderate to high yields, reaching up to 91% for the carbonylative Suzuki coupling and 94% for the carbonylative Hiyama coupling after 4–8 h at 80–90°C (Scheme 32). Moreover, both [BMIM][BF_4_] and [BMIM][PF_6_] could be recycled for up to three consecutive runs in both carbonylative protocols with only minimal erosion of product yields, further underscoring the sustainability of this NFS‐based carbonylation strategy [72].

**SCHEME 33:**
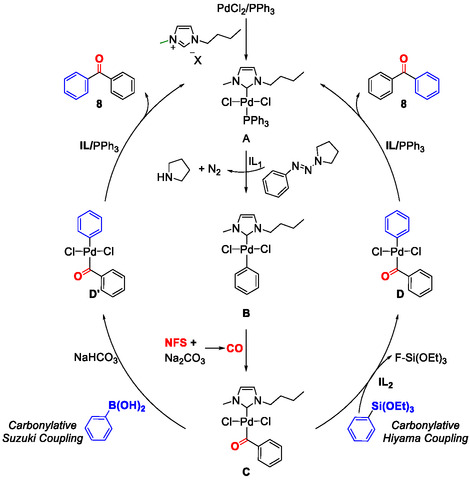
Proposed reaction mechanism for carbonylative Suzuki–Miyaura and carbonylative Hiyama reactions.

For both the carbonylative Suzuki–Miyaura and carbonylative Hiyama reactions, the mechanism depicted in Scheme 33 was proposed. It involves the in situ generation of an *N*‐heterocyclic carbene (NHC)–palladium complex **A** (NHC–PdCl_2_/PPh_3_), which could be formed through the interaction of the PdCl_2_/PPh_3_ precatalyst with the imidazolium‐based ionic liquids [BMIM][BF_4_] or [BMIM][PF_6_]. According to the proposed mechanism, IL_1_ plays a crucial role in both carbonylative couplings to demask the triazene, thereby promoting the formation of the key aryl–palladium intermediate **B**. In contrast, IL_2_ is required exclusively in the carbonylative Hiyama coupling, where it could act as a fluoride source to activate triethoxy(phenyl)silane toward transmetalation (Scheme 33) [72].

Overall, this study further highlights the versatility and sustainability of NFS‐based CO delivery in combination with green reaction media for palladium‐catalyzed carbonylative cross‐coupling reactions.

### 3.2 COgen, SilaCOgen, and Formic Acid as Ex Situ CO Sources

Although the in situ generation of CO from surrogates is widely adopted in synthetic carbonylative chemistry, the concomitant formation of byproducts derived from the CO‐releasing reagents can complicate reaction workup and product purification, thereby limiting the broader applicability of such methodologies [18, 21].

**FIGURE 1:**
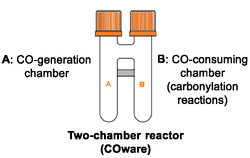
Ex situ production of CO in a two‐chamber reactor.

To circumvent these drawbacks, Skrydstrup and coworkers introduced an alternative strategy for the delivery of carbon monoxide based on a two‐chamber reactor system (also referred to as COware). In this setup, CO is generated ex situ in a dedicated chamber through the decomposition of a suitable CO surrogate, whereas the carbonylative transformation proceeds in the second, spatially separated chamber (Figure 1) [74].

**FIGURE 2:**
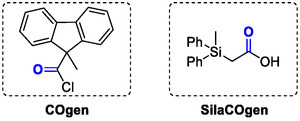
COgen and SilaCOgen.

This ex situ delivery technique has been successfully applied to a wide range of Pd‐catalyzed carbonylation protocols and is compatible with several solid CO sources, including 9‐methylfluorene‐9‐carbonyl chloride (COgen) and methyldiphenylsilacarboxylic acid (SilaCOgen) (Figure 2) [74].

COgen (Figure 2) is a bench stable, easy to manipulate and crystalline acid chloride that releases gaseous CO by a catalytic decarbonylation performed in a variety of aprotic solvents in the presence of a Pd‐based catalyst, a suitable ligand and an amine base. Upon heating to temperatures ≥80°C, COgen decomposes nearly quantitatively in the CO‐generation chamber (A) of the two‐chamber apparatus, releasing gaseous CO; simultaneously, 9‐methylene‐9*H*‐fluorene is generated as nonvolatile side product which remains confined to Chamber A and does not contaminate the CO‐consuming chamber (B) [74].

SilaCOgen (Figure 2) also constitutes a highly crystalline CO surrogate that is readily subjected to decarbonylation. In this case, CO is liberated upon treatment with a fluoride source in a range of organic solvents. Compared to COgen, SilaCOgen can effectively and rapidly release CO even at temperatures below 40°C. Both reagents have been successfully applied across a variety of carbonylative transformations conducted in the two‐chamber apparatus [74].

The first sustainable carbonylation protocol employing COgen as a bench‐stable solid precursor of carbon monoxide and anisole as an environmentally benign solvent was reported by Skrydstrup and coworkers in 2013 for the performance of carbonylative Suzuki–Miyaura couplings [75]. Using a two‐chamber reactor, thereby circumventing the direct handling of CO and enabling air‐tolerant conditions that simplify the reaction setup, the authors achieved carbonylative cross‐couplings between (hetero)aryl iodides and aryl boronic acids to deliver the corresponding benzophenone derivatives **8** in good to excellent yields (50%–93%) and with broad functional‐group compatibility.

**SCHEME 34:**
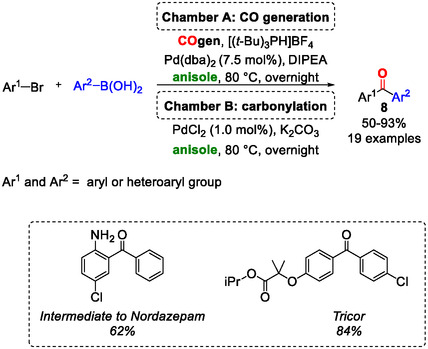
Synthesis of diaryl ketones **8** employing COgen as ex situ CO source and anisole as green solvent. DIPEA = *N*,*N*‐diisopropylethylamine; Pd(dba)_2_ = bis(dibenzylideneacetone)palladium(0).

In this setup, the first chamber contained COgen in combination with the reagents required for CO release, namely (*t*‐Bu)_3_PH]BF_4_, Pd(dba)_2_, and DIPEA, using anisole as the solvent. The second chamber was charged with the substrates and catalytic system for the Suzuki–Miyaura carbonylation, comprising the aryl halide, aryl boronic acid, PdCl_2_, K_2_(CO)_3_ and anisole (Scheme 34) [75].

In addition, the substitution of the CO surrogate with its ^13^C‐labeled version led to the corresponding ^13^C‐labeled diaryl ketone **8**.

The synthetic utility of this methodology was further illustrated by its application to the preparation of two biologically relevant targets, including a synthetic intermediate to Nordazepam, a benzodiazepine used for the treatment of anxiety disorders and Tricor, a lipid‐lowering agent, obtained in 62% and 84% yield, respectively (Scheme 34) [75].

Among renewable approaches to carbonylation chemistry, Bayer and coworkers reported in 2020 a palladium‐catalyzed carbonylative cross‐coupling strategy in which aryl bromides reacted with a variety of nucleophiles, including amines, alcohols, and aryl boronic acids. Notably, the reactions were conducted using COgen as a bench‐stable and solid CO‐releasing reagent, while a range of renewable solvents were employed as the green reaction medium, highlighting the sustainability of the overall methodology [76].

Particularly, carbonylative cross‐coupling reactions of aryl bromides with aryl boronic acids, as well as aminocarbonylation and alkoxycarbonylation processes, were carried out in two‐chamber reactor systems, employing stoichiometric amounts of carbon monoxide generated ex situ from COgen.

**SCHEME 35:**
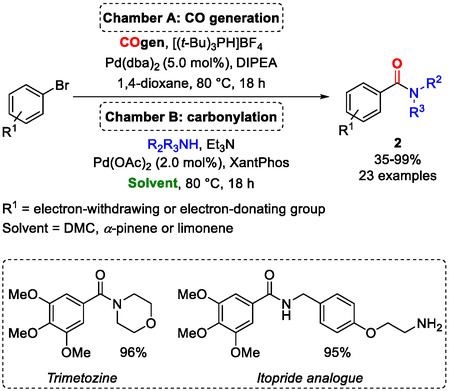
Aminocarbonylation of aryl bromides performed in green reaction media and with COgen as solid source of carbon monoxide. DMC = dimethyl carbonate; XantPhos = 4,5‐bis(diphenylphosphino)‐9,9‐dimethylxanthene.

The palladium‐catalyzed aminocarbonylation of aryl bromides was investigated in a range of renewable solvents, using a Pd(OAc)_2_/XantPhos catalytic system. Optimal performance was observed in nonpolar hydrocarbon solvents, such as α‐pinene and limonene, as well as in the nonpolar carbonate dimethyl carbonate (DMC). These environmentally benign media delivered comparable outcomes across a series of aminocarbonylation reactions, affording the corresponding amides **2** in moderate to excellent yields (35%–99%) and exhibiting broad functional‐group tolerance (such as CHO, CN, and COOMe on the aryl bromide) under the applied conditions (Scheme 35) [76].

The aminocarbonylation protocol was further applied to the synthesis of the commercially available sedative trimetozine and an analog of Itopride, a drug used in the treatment of gastrointestinal disorders. In both cases, the target compounds were obtained in excellent yields (96% and 95%, respectively), underscoring the applicability of the methodology to pharmaceutically relevant molecules (Scheme 35).

**SCHEME 36:**
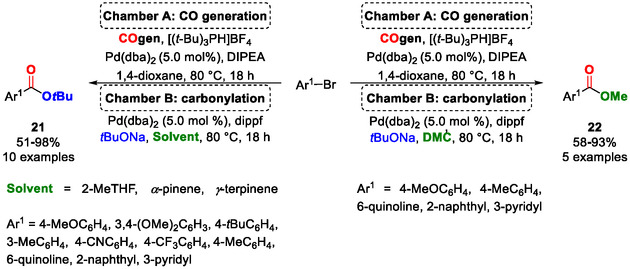
Alkoxycarbonylation of aryl bromides performed in green reaction media and with COgen as solid source of carbon monoxide. DIPEA = *N*,*N*‐diisopropylethylamine; DMC = dimethyl carbonate; dppf = 1,1′‐bis(diisopropylphosphino)ferrocene; Pd(dba)_2_ = palladium(0) bis(dibenzylideneacetone).

In addition, sustainable solvents, the carbon monoxide surrogate COgen, and a palladium‐based catalytic system consisting of Pd(dba)_2_ as the precatalyst and dippf [1,1′‐bis(diisopropylphosphino)ferrocene] as the ligand were employed to promote alkoxycarbonylation reactions. The carbonylative coupling of aromatic bromides with sodium *tert*‐butoxide as the nucleophile proceeded efficiently in renewable solvents such as α‐pinene, γ‐terpinene, and 2‐methyltetrahydrofuran (2‐MeTHF). The optimal choice of green solvent is substrate‐dependent; indeed, depending on the nature of the aryl bromide substrate, the reactions afforded the corresponding ester products **21** in yields of up to 98% (Scheme 36) [76].

Furthermore, the use of dimethyl carbonate (DMC) as an environmentally benign solvent in the alkoxycarbonylation protocol led to the formation of the corresponding methyl ester **22** (Scheme 36). This product may arise either from a Pd‐catalyzed carbonylative coupling of the aryl halide with in situ generated sodium methoxide or from a subsequent transesterification of the initially formed *tert*‐butyl ester mediated by sodium methoxide [76].

**SCHEME 37:**
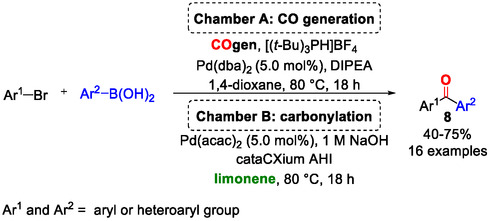
Carbonylative C–C cross‐coupling in limonene as green reaction medium and COgen as solid source of carbon monoxide. cataCXium AHI = di(1‐adamantyl)*‐N*‐butylphosphine hydroiodide; DIPEA = *N*,*N*‐diisopropylethylamine; Pd(acac)_2_ = palladium(II) acetylacetonate; Pd(dba)_2_ = bis(dibenzylideneacetone)palladium(0).

Moreover, by screening a range of renewable solvents, including α‐pinene, limonene, γ‐terpinene, DMC, and 2‐MeTHF, among others, the authors demonstrated that the carbonylative synthesis of unsymmetrical ketones from aryl bromides and aryl boronic acids proceeded most efficiently in limonene (Scheme 37). Both electron‐rich and electron‐deficient aromatic and heteroaromatic boronic acids, as well as a variety of aromatic bromides, were well tolerated, affording the corresponding ketones **8** in moderate to good yields, ranging from 40% to 75% (Scheme 37) [76].

**SCHEME 38:**
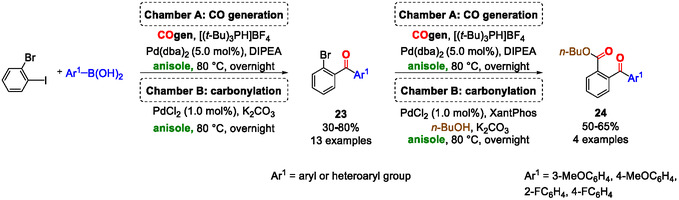
Sustainable synthesis of 2‐aroylbenzoate esters **24** by carbonylative Suzuki–Miyaura coupling of 2‐bromoiodobenzene with aryl boronic acids followed by palladium‐catalyzed alkoxycarbonylation of 2‐bromo biaryl ketones **23**. DIPEA = *N*,*N*‐diisopropylethylamine; Pd(dba)_2_ = bis(dibenzylideneacetone)palladium(0); XantPhos = 4,5‐bis(diphenylphosphino)‐9,9‐dimethylxanthene.

**SCHEME 39:**
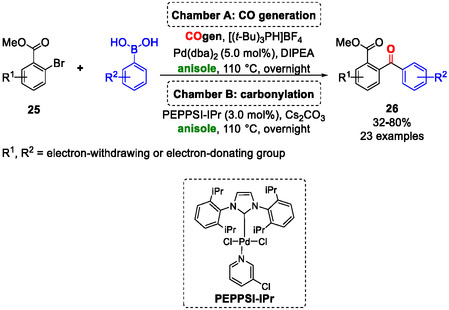
Synthesis of 2‐aroylbenzoate esters **26** by carbonylative Suzuki–Miyaura coupling between methyl 2‐bromobenzoates **25** and boronic acid. DIPEA = *N*,*N*‐diisopropylethylamine; Pd(dba)_2_ = bis(dibenzylideneacetone)palladium(0).

In 2020, the same authors reported two alternative carbonylative strategies for the synthesis of diversely substituted diaryl ketones in the form of 2‐aroylbenzoate esters (**24** and **26** in Schemes 38 and 39), which represent valuable synthetic intermediates for the construction of biologically active molecules [77, 78]. In both methodologies, anisole was employed as an environmentally benign solvent, while COgen served as a safe, convenient, and readily handled ex situ source of carbon monoxide in a sealed two‐chamber system (COware) [79]. In the first approach (Scheme 38), functionalized esters **24** were obtained through a two‐step sequence. Initially, a palladium‐catalyzed carbonylative Suzuki–Miyaura cross‐coupling between 2‐bromoiodobenzene and a range of (hetero)aryl boronic acids afforded substituted 2‐bromobiaryl ketones **23**.

These intermediates were subsequently subjected to a Pd‐catalyzed alkoxycarbonylation reaction in the presence of *n*‐butanol as the nucleophile, delivering the corresponding esters **24** in moderate yields (50%–65%).

However, while the carbonylative Suzuki–Miyaura coupling proved to be broadly applicable, enabling the synthesis of a variety of ketones **23**, the subsequent alkoxycarbonylation step exhibited limited substrate scope. In particular, the efficiency of this transformation was strongly dependent on the substitution pattern of the ketone precursors **23**, thereby restricting access to a wide library of esters **24** (Scheme 38) [79].

In light of the limited substrate scope observed for the alkoxycarbonylation of 2‐bromobiaryl ketones **23**, for the preparation of 2‐aroylbenzoate esters, the authors subsequently explored an alternative carbonylative Suzuki–Miyaura coupling between methyl 2‐bromobenzoates **25** and aryl boronic acid derivatives (Scheme 39).

This second strategy exhibited a significantly broader substrate scope. A wide range of both electron‐rich and electron‐poor aryl boronic acids, as well as methyl 2‐bromobenzoates **25** bearing either electron‐donating or electron‐withdrawing substituents, were well tolerated under the optimized conditions. As a result, a diverse set of diaryl ketone derivatives **26** was obtained in moderate to good yields (32%–80%, Scheme 39) [79]. Notably, in both carbonylative protocols, the slow addition of the boronic acid during the Suzuki–Miyaura coupling was found to favor the formation of the desired carbonylative product over the formation of the noncarbonylative side product, leading to improved yields.

Palladium‐catalyzed carbonylation reactions constitute a powerful class of transformations that enable access to a wide variety of carbonyl‐containing molecules. However, palladium‐catalyzed thiocarbonylations have remained comparatively underexplored. This is likely attributable to the propensity of thiol nucleophiles to poison palladium catalysts. Nevertheless, several sustainable carbonylative procedures for the synthesis of thioesters, valuable acyl donors widely employed in organic synthesis [80, 81, 82], have been reported.

**SCHEME 40:**
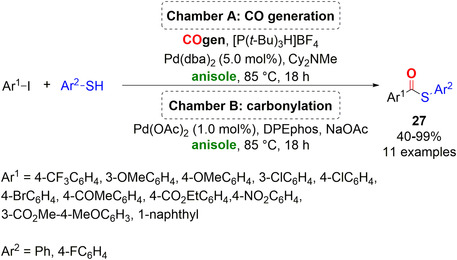
Pd‐catalyzed thiocarbonylation of electron‐deficient aryl iodides employing COgen as CO precursor and anisole as green reaction medium. DPEphos = bis[(2‐diphenylphosphino)phenyl] ether; Pd(dba)_2_ = bis(dibenzylideneacetone)palladium(0).

For example, the two‐chamber system employing the acid chloride‐based precursor COgen for the ex situ generation of CO, together with anisole as a green solvent, also has proven effective for thioester formation. In particular, Skrydstrup's group developed a Pd‐catalyzed thiocarbonylative coupling of aryl iodides with aromatic thiols using stoichiometric carbon monoxide (Scheme 40) [83]. Both electron‐rich and electron‐poor aryl iodides were compatible with the protocol, although the reaction outcome was shown to depend on the reaction solvent (DME or anisole). Specifically, for electron‐rich aryl iodides, optimal conditions consisted of Pd(OAc)_2_ (1.0 mol%) in combination with DPEphos [bis[(2‐diphenylphosphino)phenyl] ether] as the ligand, NaOAc as the base, and DME as the solvent, affording the desired thiocarbonylation products in good yields. In contrast, when electron‐deficient aryl iodides were employed, the best performance was achieved using Pd(OAc)_2_/DPEphos as the catalytic system with NaOAc as the base, in anisole as a sustainable reaction medium. Under the optimized conditions, the corresponding thioesters **27** were obtained in yields of up to 99%, while minimizing the formation of the competing noncarbonylated thioether byproduct (Scheme 40) [83].

**SCHEME 41:**
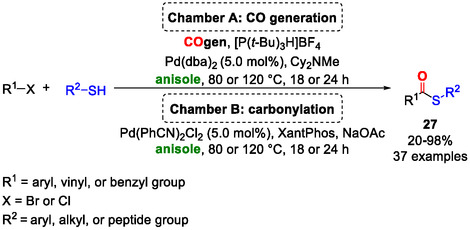
Pd‐catalyzed thiocarbonylation of aryl, vinyl, and benzyl halides using COgen as CO precursor and anisole as sustainable solvent. DPEphos = bis[(2‐diphenylphosphino)phenyl]ether; Pd(dba)_2_ = bis(dibenzylideneacetone)palladium(0); XantPhos = 4,5‐bis(diphenylphosphino)‐9,9‐dimethylxanthene.

Subsequently, the same group extended carbonylative protocol to the more synthetically attractive aryl, vinyl, and benzyl bromides as well as benzyl chlorides, for the corresponding thioester synthesis [84]. In this study, a catalytic system composed of bis(benzonitrile)palladium(II) chloride and XantPhos as the ligand, together with NaOAc as a weak base, stoichiometric CO generated from COgen in a two‐chamber setup, and anisole as the solvent at 120°C, proved to be effective for the Pd‐catalyzed thiocarbonylation of electron‐rich aryl bromides, affording the desired thioesters **27** in yields of up to 98% (Scheme 41) [84].

When electron‐deficient aryl bromides were employed, the same reaction conditions led to decreased yields and lower chemoselectivity due to the competing formation of thioether byproducts. To improve selectivity toward thioester formation and increase yields up to 80%, the addition of NaI was found to be crucial. The effect of iodide was rationalized by the formation of Pd–I complexes via bromide displacement, thereby facilitating the CO insertion step into the Ar—Pd bond during the catalytic cycle.

The substrate scope of this Pd‐catalyzed thiocarbonylation also included vinyl and benzyl bromides, as well as benzyl chlorides. In these cases, reactions were generally conducted at reduced temperature (80°C) to suppress competitive thioether formation and favor the desired thioester products (Scheme 41) [84].

COgen is considered advantageous as an ex situ CO‐releasing reagent for carbonylation reactions owing to its high crystallinity and ease of handling. However, CO liberation requires a Pd‐catalyzed decarbonylation process that operates at elevated temperatures, which may limit its broader applicability.

**SCHEME 42:**
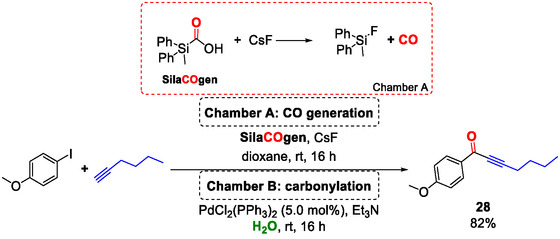
Pd‐catalyzed carbonylative Sonogashira of 1‐iodo‐4‐methoxybenzene with 1‐hexyne performed in aqueous medium and SilaCOgen as CO surrogate.

To address these drawbacks, Skrydstrup and coworkers developed an alternative CO surrogate based on a silacarboxylic acid, termed SilaCOgen (Scheme 42) [85]. This compound was synthesized in 77% overall yield from chloro(methyl)diphenylsilane through a two‐step sequence consisting of lithium metal reduction followed by carboxylation with carbon dioxide [85].

SilaCOgen proved to be an air‐stable and user‐friendly CO precursor that undergoes rapid decarbonylation under metal‐free conditions and at low temperature. Owing to the strong fluorophilicity of silicon, CO release can be triggered by treatment with a fluoride activator such as KF or CsF at, or near, room temperature. When employed in near‐stoichiometric amounts, SilaCOgen enabled a variety of Pd‐catalyzed carbonylative couplings in the two‐chamber reactor system. As a representative example, it proved highly effective in the carbonylative Sonogashira coupling of 1‐iodo‐4‐methoxybenzene with 1‐hexyne, affording the corresponding alkynone **28** in 82% yield (Scheme 42). The carbonylation reaction was carried out at room temperature in water using PdCl_2_(PPh_3_)_2_ as the catalyst (Chamber B, Scheme 42). However, CO release from SilaCOgen in aqueous medium was not optimal, likely due to hydration of the fluoride anion and the resulting decrease in nucleophilicity. Consequently, the precursor was dissolved in dioxane, and CsF was employed as the fluoride source to ensure efficient CO generation (Chamber A, Scheme 42 [85]).

Beyond COgen and SilaCOgen as ex situ CO‐releasing molecules, the two‐chamber setup also enables the use of other CO surrogates, such as formic acid [18, 21].

**SCHEME 43:**
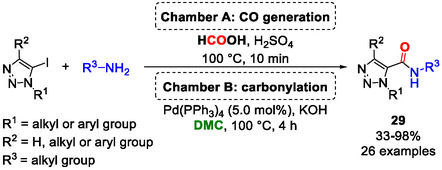
Synthesis of 1,2,3‐triazole‐5‐carboxamides **29**, using dimethyl carbonate as sustainable solvent and formic acid as ex situ CO‐releasing molecule. DMC = dimethyl carbonate.

In this context, in 2019, Schwab and coworkers established a simple Pd‐catalyzed aminocarbonylation reaction of 5‐iodo‐1,2,3‐triazoles with amines, in which carbon monoxide was generated ex situ by thermal decomposition of formic acid in the presence of sulfuric acid at 100°C (Chamber A, Scheme 43) [86]. Owing to its advantageous properties, such as high biodegradability, low toxicity, and peculiar reactivity, the carbonylative cross‐coupling (Chamber B) proceeded in dimethyl carbonate (DMC) as an environmentally benign solvent [48], using the palladium(0) catalyst Pd(PPh_3_)_4_ without any additional ligand, and KOH as base. After 4 h at 100°C, trisubstituted 1,2,3‐triazole‐5‐carboxamides **29**, a class of highly valuable heterocycles [87, 88], were obtained in yields up to 98% (Scheme 43). The optimized conditions tolerated a broad range of 5‐iodo‐1,4‐disubstituted‐1,2,3‐triazoles, including substrates bearing biologically relevant motifs (e.g., sugar, cholesterol, and chalcone fragments) on C‐4, as well as several primary aliphatic amines. In contrast, secondary and aromatic amines such as diethylamine, piperidine, and aniline were unreactive, presumably due to their reduced nucleophilicity for steric or electronic reasons and/or decreased ability to coordinate palladium [86].

## 4 Summary and Outlook

We have summarized the most recent advances in the field of green carbonylation reactions, highlighting the progress achieved through the combined use of safer CO surrogates and sustainable solvents. The methodologies discussed involved a wide range of organic substrates such as amines, alcohols, thiols, selenols, boronic acids, and alkynes that were efficiently coupled with aryl halides, vinyl halides, and even aryl diazonium salts, thus enabling access to a wide range of molecular architectures. Importantly, the use of green, safe, and renewable solvents did not compromise the applicability of the reactions and, in some instances, even enabled new and intriguing reactivity pathways.

Despite these significant developments, several fundamental challenges remain to be addressed in order to render carbonylation methodologies fully aligned with the principles of green chemistry and suitable for large‐scale application.

With respect to the carbonyl source, the identification and implementation of nontoxic, organic CO surrogates that comply with the principle of atom economy will be of paramount importance. Among the various candidates, carbon dioxide stands out as an ideal C1 building block due to its abundance, low cost, and renewable nature. The direct or indirect utilization of CO_2_ as a carbonylating agent would not only reduce reliance on gaseous CO but also contribute to carbon capture and valorization strategies

Among the limited number of methodologies reported for the reduction of CO_2_ to CO and its subsequent application in the synthesis of carbonyl compounds, of particular note are the following methodologies: (1) a formal Rh‐catalyzed hydroxycarboxylation of alkenes, resulting from a combination of Rh‐catalyzed reverse water–gas shift reduction of CO_2_ to CO and subsequent Rh‐catalyzed carbonylation process to obtain carboxylic acids [89, 90]; (2) an Rh‐catalyzed alkoxycarbonylation of alkenes, in which carbon dioxide is reduced to carbon monoxide by the alcohol via a “hydrogen‐borrowing” process [91]; (3) an Rh‐catalyzed hydroformylation of olefins that affords aldehydes through a tandem sequence of poly(methylhydrosiloxane)‐mediated CO_2_ reduction to CO and a conventional Rh‐catalyzed hydroformylation step with CO/H_2_ [92]; (4) a fluoride‐catalyzed deoxygenation of CO_2_ to CO, in the presence of disilanes, followed by a conventional amino‐ or alkoxycarbonylation reaction mediated by Pd [93]; (5) an electrochemical reduction of CO_2_, mediated by an iron‐tetraphenylporphyrinato complex as electron source, coupled with a standard Pd‐catalyzed carbonylation for the synthesis of amides, esters and ketones [94].

**SCHEME 44:**
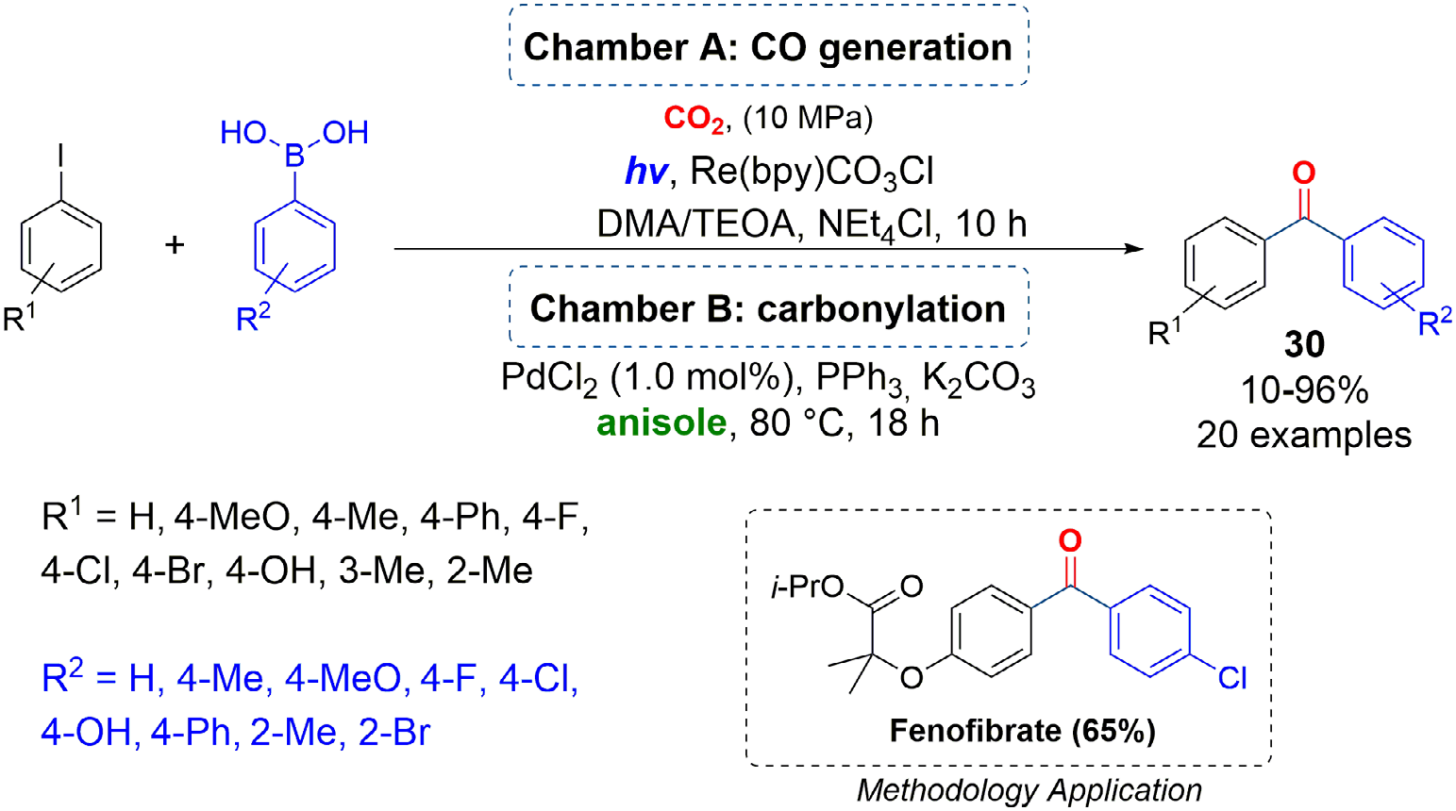
Synthesis of diaryl ketones **30** by sequential Re‐catalyzed photoreduction of CO_2_ to CO and Pd‐catalyzed carbonylative coupling between aryl iodides and aryl boronic acids. DMA = *N*,*N*‐dimethylacetamide; TEOA = triethanolamine.

Within the context of synthetic applications of CO generated by photochemical CO_2_ reduction, in 2018, He and coworkers described a functionalization of CO_2_ by combing the rhenium photocatalysis with the palladium chemistry for the carbonylative coupling of aryl iodides with arylboronic acids, in anisole as sustainable medium (Scheme 44) [95].

The methodology relies on a two‐autoclave setup in which CO is generated in situ via the photoreduction of CO_2_ (0.2 MPa) under irradiation of a Re(I) bipyridine tricarbonyl complex with a 500 W high‐pressure mercury arc lamp. The resulting CO is subsequently channeled to a second autoclave, where PdCl_2_ catalyzes the carbonylative coupling of aryl iodides with aryl boronic acids, affording a range of diversely functionalized benzophenone derivatives **30**.

When testing the range of applicability of the method the authors found that both electron‐rich and electron‐poor aryl iodides were well tolerated, while steric hindrance emerged as a key limiting factor when ortho substituted reagents were employed. Notably, the method accommodated sensitive functional groups such as free phenols. In contrast, aryl boronic acids bearing electron‐withdrawing substituents performed better than their electron‐donating counterparts, which afforded the carbonylated products in only moderate yields. In line with the observed substrate generality, the industrially relevant, hypolipidemic agent, fenofibrate was successfully synthesized in 65% yield, further underscoring the practical feasibility of the protocol and its potential to expand the scope of CO_2_‐based, value‐added transformations. Finally, to confirm the role of CO_2_ as the source of CO, an experiment with ^13^CO_2_ was performed, leading to the formation of the expected ^13^C‐labeled benzophenone. One aspect of the methodology that could be improved from a sustainability perspective is the reaction medium, consisting of a mixture of dimethylacetamide and triethanolamine, in which the CO_2_ photoreduction is carried out.

**SCHEME 45:**
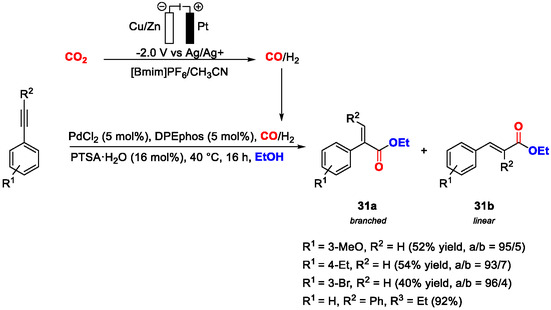
Synthesis of branched and linear ethyl acrylates **31a,b** by combining the electroreduction of CO_2_ to CO with conventional Pd‐catalyzed alkoxycarbonylation reaction in ethanol.

A promising strategy for the improvement of carbonylation chemistry sustainability involves the electroreduction of CO_2_ to cleanly produce CO as recently demonstrated by Huang and Dong [96]. In 2024, they found that internal alkynes could be regioselectively transformed in branched (**31a**) or linear (**31b**) acrylates by combining the reduction of CO_2_ to CO with conventional Pd‐catalyzed alkoxycarbonylation reaction (Scheme 45). Electrolysis was performed in a double‐compartment cell at a constant potential (−2.0 V vs. Ag/Ag^+^) using a Cu/Zn cathode and a platinum gauze anode, with CO_2_ flowing into the electrochemical reaction cell at a flow rate of 1.4–1.6 mL min^−1^. After 3 h of electrolysis, a gas mixture of CO_2_ (61 %vol), CO (16 %vol), and H_2_ (23 %vol) was produced, collected in a gas bag, and then subjected into a separate reactor for the Pd‐catalyzed alkoxycarbonylation step. The synthetic methodology was successfully extended to a wide range of both internal and terminal alkynes, which were efficiently coupled with primary alcohols, with methanol being, unfortunately, the most extensively investigated substrate.

In line with the scope and sustainability‐oriented focus of this review, only the transformations conducted in green media ethanol have been highlighted (Scheme 45).

Furthermore, although the methodology exhibits a high degree of sustainability compared to conventional carbonylation processes, it could be further improved by employing a reaction medium alternative to acetonitrile in the CO_2_ electroreduction step.

Finally, a major objective for the future advancement of sustainable carbonylation chemistry is the transition from catalysts based on scarce noble metals, such as Pd and Rh, toward systems relying on earth‐abundant metals. Although palladium‐based catalysts have demonstrated unmatched versatility and efficiency in carbonylation chemistry, their limited availability, high cost, and environmental footprint pose intrinsic sustainability concerns. In this context, the development of catalytic systems based on iron and copper could represent a highly attractive direction for future applications.

Overall, the future of green carbonylation chemistry will likely rely on the convergence of many strategies: the transition from noble to earth‐abundant metals, the adoption of green solvents and nontoxic carbonyl sources such as CO_2_, and the exploitation of renewable energy inputs. Achieving sustainability in chemical processes is not a matter of single‐step solutions, but a continuous, complex endeavor that calls for holistic strategies and an integrated view of the entire system.

## Conflicts of Interest

The authors declare no conflicts of interest.

## Data Availability

The data that supports the findings of this study are available in the Supporting Information of this article.
